# Apelin Rejuvenates Aged Human Mesenchymal Stem Cells by Regulating Autophagy and Improves Cardiac Protection After Infarction

**DOI:** 10.3389/fcell.2021.628463

**Published:** 2021-03-02

**Authors:** Hao Zhang, Chengling Zhao, Guojun Jiang, Bei Hu, Huifeng Zheng, Yimei Hong, Zhen Cui, Linli Shi, Xin Li, Fang Lin, Yue Ding, Lu Wei, Mimi Li, Xiaoting Liang, Yuelin Zhang

**Affiliations:** ^1^Faculty of Pharmacy, Bengbu Medical College, Bengbu, China; ^2^Department of Emergency Medicine, Department of Emergency and Critical Care Medicine, Guangdong Provincial People’s Hospital, Guangdong Academy of Medical Sciences, Guangzhou, China; ^3^Department of Respiratory Medicine, The First Affiliated Hospital of Bengbu Medical College, Bengbu, China; ^4^Department of Radiation Oncology, The First Affiliated Hospital of Bengbu Medical College, Bengbu, China; ^5^Institute of Regenerative Medicine, Shanghai East Hospital, Tongji University School of Medicine, Shanghai, China; ^6^Clinical Translational Medical Research Center, Shanghai East Hospital, Tongji University School of Medicine, Shanghai, China; ^7^Department of Organ Transplantation, Changzheng Hospital, Second Military Medical University, Shanghai, China

**Keywords:** apelin, mesenchymal stem cells, rejuvenation, senescence, myocardial infarction

## Abstract

The protective effects of mesenchymal stem cell (MSC)-based therapy for myocardial infarction (MI) are largely hampered as they age. Apelin is an endogenous ligand of its receptor APJ and plays an essential role in regulating multiple biological activities including MSC proliferation and survival. In this study, we investigated whether Apelin regulates MSC senescence and whether its overexpression could rejuvenate aged MSCs (AMSCs) to improve cardiac protection following infarction in mice. MSC senescence was evaluated by senescence-associated β-galactosidase assays. Apelin level was examined by western blotting. Autophagy was determined by transmission electron microscopy. The cardioprotective effect of AMSCs with Apelin overexpression (Apelin-AMSCs) was assessed in a mouse MI model. Apelin expression was dramatically reduced in AMSCs. Interestingly, knockdown of Apelin induced young MSCs (YMSC) senescence, whereas overexpression rescued AMSC senescence. Apelin overexpression also increased AMSC angiogenic capacity. Mechanistically, Apelin overexpression upregulated the autophagy level of AMSCs by activating AMP-activated protein kinase (AMPK) signaling, thereby rejuvenating AMSCs. Compared with AMSCs, transplantation of Apelin-AMSCs achieved better therapeutic efficacy for MI by enhancing cell survival and angiogenesis. In conclusion, our results reveal that Apelin activates AMPK to rejuvenate AMSCs by increasing autophagy and promotes cardioprotection following infarction in mice. This study identified a novel target to rejuvenate AMSCs and enhance their therapeutic efficacy.

## Introduction

Myocardial infarction (MI) is a leading cause of morbidity and mortality worldwide in older adults. Given the growing aging population with a number of potential risk factors, MI prevalence is expected to increase. Despite significant progress in pharmacological and surgical treatments, existing therapies are not sufficient to improve the clinical outcomes of MI. Preclinical studies and clinical trials in the last decade have demonstrated that mesenchymal stem cells (MSC)-based therapy is a novel strategy for MI treatment due to easy isolation, multilineage differentiation, and low risk of immune rejection (Zhang et al., 2017; Xiao et al., 2018; Liao et al., 2019). However, the functions of MSCs isolated from aged patients dramatically decline, undoubtedly reducing their therapeutic efficacy for MI (Zhang et al., 2019; Sun et al., 2020). There is an urgent need to rejuvenate aged-MSCs (AMSCs) to improve their beneficial effects. Some strategies, including genetic modification and pharmacological pretreatment, are being explored to rejuvenate AMSCs (Song et al., 2017; Liang et al., 2019; Hong et al., 2020). Understanding the molecular mechanism involved in MSC senescence is an obvious prerequisite for preventing cellular senescence and identifying new targets for rejuvenating AMSCs.

Apelin is an endogenous polypeptide ligand for the orphaned G protein-coupled receptor APJ that plays a critical role in regulating cell proliferation, apoptosis, and migration (Chapman et al., 2014). Activation of the apelin/APJ pathway induces diverse physiological effects including angiogenesis, cardiovascular functions, fluid homeostasis and energy metabolism regulation (Chaves-Almagro et al., 2015). Apelin has been extensively described as a beneficial adipokine regarding to glucose and lipid metabolism, with anti-diabetic and anti-obesity properties. Accumulating evidence indicates that Apelin mediates MSC differentiation, proliferation, and survival (Liang et al., 2016; Hang et al., 2019). Hypoxia promotes MSC proliferation by activating the apelin/APJ/autophagy signaling pathway, and these effects are partially abrogated by downregulation of APJ (Li et al., 2015). The therapeutic efficacy of MSCs for MI is largely attributed to their paracrine effects (Deng et al., 2020; Sid-Otmane et al., 2020). Notably, Apelin also regulates angiogenesis (Uribesalgo et al., 2019); it improves MSC vascularization under hypoxic conditions by upregulating the level of vascular endothelial growth factor (Hou et al., 2017). We previously demonstrated that the angiogenic capacity of AMSCs is greatly reduced (Zhang et al., 2019; Hong et al., 2020). These findings prompted us to investigate a possible role for Apelin in regulating MSC senescence. However, whether and how Apelin regulates MSC senescence still remains unclear.

It is well known that the autophagic level is closely associated with MSC senescence (Liu et al., 2020; Zhou et al., 2020). Compared with healthy MSCs, MSCs isolated from patients with abdominal aortic aneurysm exhibit senescence as evidenced by increased senescence-associated secretory phenotype and decreased proliferative capacity, and these effects are remarkably reversed by the autophagy activator rapamycin (Huang et al., 2019). AMSCs exhibit reduced autophagy accompanied by decreased self-renewal, lower regenerative capacity, and replicative exhaustion (Revuelta and Matheu, 2017). Since Apelin is involved in regulating autophagy (Zhu et al., 2019), we hypothesized that Apelin regulates MSC senescence in the same way. Here, we report that downregulation of Apelin affects AMP-activated protein kinase (AMPK) signaling to induce MSC senescence by inhibiting autophagy, whereas Apelin overexpression rejuvenated AMSCs and enhanced their therapeutic efficacy for MI in a mouse model.

## Materials and Methods

### Cell Culture

The bone marrow of young and aged volunteer donors were obtained with informed consent in the current study. This study was approved by the research ethics board of Shanghai East Hospital (No. 2016-050). Young-MSCs (YMSCs) and AMSCs were isolated as previously reported (Liang et al., 2019). YMSCs and AMSCs were regularly cultured in Dulbecco’s minimum essential medium (DMEM)/high glucose (Gibco, Grand Island, NY, United States; 11965084) supplemented with 10% fetal bovine serum (FBS; Life Technologies, Carlsbad, CA, United States; 16000), 5 ng/mL endothelial growth factor (PeproTech, Rocky Hill, NJ, United States: AF-100-15), and 5 ng/mL basic fibroblast growth factor (PeproTech; 100-18B) at 37°C in a humidified atmosphere with 5% CO_2_. All MSCs were used at passage 3–4 in the current study. Human umbilical vein endothelial cells (HUVECs) were cultured in RPMI 1640 (Gibco; C11875500BT) supplemented with 10% FBS.

### Senescence-Associated β-Galactosidase (SA-β-gal) Assay

Mesenchymal stem cell senescence was evaluated with SA-β-gal assay kits (Beyotime, Shanghai, China; C0602). Briefly, MSCs were seeded in 6-well plates and subjected to different treatments. Next, MSCs were fixed for 20 min, washed with phosphate-buffered saline (PBS), and incubated with the SA-β-gal labeling solution overnight at 37°C without CO_2_. Finally, SA-β-gal-positive cells were randomly photographed and counted. The percentage of senescent MSCs was calculated as the ratio of SA-β-gal-positive MSCs to the total number of MSCs obtained from five different fields of view.

### Enzyme-Linked Immunosorbent Assay

The concentration of secreted Apelin in conditioned media (CdM) from YMSCs and AMSCs was determined with Apelin enzyme-linked immunosorbent assay (ELISA) kits according to the manufacturer’s protocol (G-Biosciences, New Delhi, India; IT11989).

### Small Interfering RNA Treatment

To knockdown Apelin, AMSCs were transfected with Apelin-small interfering RNA (siRNA) (Santa Cruz Biotechnology, Dallas TX, United States; sc-44741) or control siRNA (Santa Cruz; sc-37007) using a Lipofectamine RNAiMAX Reagent Kit (Invitrogen, Carlsbad, CA, United States; 13778030) according to the protocol. Transfection efficiency was examined by western blotting 72 h after transfection.

### Viral Vector Construction and Infection

The lentiviral plasmid constructs for Apelin were purchased from GenePharma (Suzhou, China). The plasmid contained an expression cassette consisting of a cytomegalovirus promoter followed by cDNA encoding mCherry and an Aplein sequence (plasmid map shown in Supplementary Figure 1). The lentivirus was packaged as previously reported (Liang et al., 2019). For stable transduction, AMSCs at a confluence of 70–80% were infected by lentivirus at a multiplicity of infection of 10 with polybrene (8 μg/mL). Infection efficiency was determined based on the mCherry fluorescent signal viewed under the microscope 48 h after infection, and positive cells were labeled as Apelin-AMSCs.

### CdM Collection

Young-MSCs and AMSC CdM was collected as previously described (Hong et al., 2020). Briefly, 5 × 10^6^ YMSCs or AMSCs were plated on a 15-cm culture dishes and cultured until they reached 70–80% confluence. Subsequently, the regular culture medium was discarded and replaced with 15 ml of serum- and antibiotic-free DMEM. Twenty-four hours later, the supernatant was gently harvested and passed through a 0.22-μm filter. Finally, the medium was centrifuged and concentrated using ultrafiltration conical tubes (Amicon Ultra-15 with membranes selective for 5 kDa).

### Tube Formation Assay

The angiogenic effect of MSC-derived CdM was assessed with capillary tube formation assays. Briefly, 3 × 10^4^ HUVECs were plated in 96-well plates coated with growth-factor-reduced Matrigel (BD Biosciences, San Jose, CA, United States; 356230). Next, HUVECs were treated with CdM from YMSCs, AMSCs, or Apelin-AMSCs for 6 h. Finally, capillary-like tube formation was randomly photographed. Tube lengths were calculated by ImageJ software (National Institutes of Health, Bethesda, MD, United States).

### Transmission Electron Microscopy

The autophagosomes of MSCs from different groups were viewed with Transmission electron microscopy (TEM). Briefly, MSCs were fixed with 2.5% glutaraldehyde in phosphate buffer for 4 h and then postfixed with 1% OsO_4_ for 2 h. Subsequently, MSCs were dehydrated with a graded concentration of ethanol (30, 50, 70, 80, 90, 95, and 100%). Next, MSCs were infiltrated with 1:1 acetone:Spurr resin (SPI-Chem, 02690-AB) for 1 h at room temperature, 1:3 acetone:Spurr resin for 3 h, and then absolute Spurr resin overnight. Finally, images were acquired using an H-7650 TEM (Hitachi, Tokyo, Japan).

### Western Blotting

Total protein from MSCs was extracted using a total protein extraction kit (Bestbio, Xi’an, China; BB-3101), and the concentrations were measured with bicinchoninic acid assay kits (Thermo Fisher Scientific, Waltham, MA, United States; 231227). A total of 30 μg protein was resolved by 10% Tris-glycine gel electrophoresis and then transferred onto polyvinylidene fluoride (PVDF) membranes. After blocking with 5% fat-free milk in Tris-buffered saline with Tween (TBST), the PVDF membranes were incubated overnight at 4°C with the following antibodies: anti-p16 (proteintech, 10883-1-AP), anti-p21 (Abcam; ab109199), anti-Apelin (Abcam; ab59469), anti-p-AMPK (CST, Danvers, MA, United States; 4184), anti-AMPK (CST; 5832), anti-LC3I/II (CST; 12741), anti-Beclin (CST; 3738), anti-p62 (CST; 5114), and anti-glyceraldehyde 3-phosphate dehydrogenase (GAPDH; CST; 2118). Next, the membranes were washed three times with TBST and incubated with secondary antibodies (1:1,000; CST) at room temperature for 1 h and then exposed in a dark room. Western blots were analyzed using ImageJ software in three independent experiments.

### MI Model Establishment and MSC Transplantation

All animal experiments were approved by the Committee on the Use of Live Animals in Teaching and Research of the Tongji University for Laboratory Animal Medicine (approval number: TJBB00120102). The MI model was established in C57/B6J mice at 6–8 weeks of age by ligating the left anterior decedent coronary artery (LAD) as previously described (Liang et al., 2019). After LAD ligation, all MI mice were intramuscularly injected with one of the following treatments: (1) PBS (MI group, *n* = 12); (2) 3 × 10^5^ YMSCs (YMSC group, *n* = 12); (3) 3 × 10^5^ AMSCs (AMSC group, *n* = 12) or 3 × 10^5^ Apelin-AMSCs (Apelin-AMSC group, *n* = 12) at four sites around the border zone of the infarcted heart. All MSCs were suspended in 40 μL PBS. The mice that underwent thoracotomy without LAD ligation served as the sham group (Sham group, *n* = 6). Cardiac function in the different groups was measured using transthoracic echocardiography (Ultramark 9; Soma Technology, Bloomfield, CT, United States) at baseline (before MI) and 1 and 28 days following MI.

### Masson’s Staining

After measuring heart function at 28 days post-MI, all mice were sacrificed, and heart tissues were harvested. After paraffin embedding, the hearts were cut into 5-μm sections. Next, Masson’s staining was performed to detect fibrosis according to the manufacturer’s protocol (Sigma, St. Louis, MO, United States; HT15). Finally, the percentage of infarct size was evaluated as the ratio of fibrosis area to total left ventricular area × 100%.

### Immunofluorescence Labeling

To examine capillaries and small arteries at day 28 post-MI, heart sections were stained with anti-CD31 (Abcam; ab19898) and anti-α-smooth muscle actin (α-SMA) (Abcam; ab5694), respectively. Capillary and arteriole densities were evaluated using the average number of CD31- or α-SMA-positive blood vessels per field (×100).

### Polymerase Chain Reaction

The expression of the senescence-associated secretory phenotype (SASP) including *matrix metallopeptidase 3 (MMP3), interleukin 1 beta (IL-1β), C-C motif chemokine ligand 5 (RANTES) and tumor necrosis factor alpha (TNF-*α) expression, and angiogenesis cytokines including *fibroblast growth factor 2 (FGF2), heparin binding EGF like growth facto (HBEGF), hepatocyte growth factor (HGF), and insulin like growth factor (IGF)* were determined by quantitative real-time Polymerase chain reaction (PCR). Briefly, total RNA was extracted from skin specimens with RNeasy Mini Kit (Qiagen, 74124). cDNA was synthesized from 500 ng of total RNA with a RevertAid First Strand cDNA Synthesis Kit (Takara, RR0036A). Then quantitative reverse transcription polymerase chain reaction analysis was performed with Fast SYBR Green Master Mix (4385617) in an ABI QuantStudio 6 Flex System. The relative standard curve method (2^–Δ^ ^Δ^ CT) was used to determine the relative mRNA expression, with *glyceraldehyde 3-phosphate dehydrogenase (GAPDH)* as the reference gene. The primers were listed in Table 1. *Human Alu-sx* repeat sequences in heart tissue from the different groups was evaluated by genomic PCR as previously described (Deng et al., 2020). The *human Alu-sx* primers were: F:5′-GGCGCGGTGGCTCACG-3′, R:5′-TTTTTTGAGACGGAGTCTCGCTC-3′. The *human GAPDH* primers were: F:5′-GTCTCCTCTGACTTCAACAGCG-3′, R:5′-ACCACCCTGTTGCTGTAGCCAA-3′. The product was detected by electrophoresis in 1.5% agarose gel supplemented with ethidium bromide.

**TABLE 1:** Primer sequence.

Gene	Sequence
IL-1β-forward	CCACAGACCTTCCAGGAGAATG
IL-1β-reverse	GTGCAGTTCAGTGATCGTACAGG
Rantes-forward	CCTGCTGCTTTGCCTACATTGC
Rantes-reverse	ACACACTTGGCGGTTCTTTCGG
TNF-α-forward	CTCTTCTGCCTGCTGCACTTTG
TNF-α-reverse	ATGGGCTACAGGCTTGTCACTC
MMP3-forward	CACTCACAGACCTGACTCGGTT
MMP3-reverse	AAGCAGGATCACAGTTGGCTGG
FGF2-forward	AGCGGCTGTACTGCAAAAACGG
FGF2-reverse	CCTTTGATAGACACAACTCCTCTC
HBEGF-forward	TGTATCCACGGACCAGCTGCTA
HBEGF-reverse	TGCTCCTCCTTGTTTGGTGTGG
HGF-forward	GAGAGTTGGGTTCTTACTGCACG
HGF-reverse	CTCATCTCCTCTTCCGTGGACA
IGF-forward	CTCTTCAGTTCGTGTGTGGAGAC
IGF-reverse	CAGCCTCCTTAGATCACAGCTC
GAPDH forward	GTCTCCTCTGACTTCAACAGCG
GAPDH reverse	ACCACCCTGTTGCTGTAGCCAA

### Statistical Analysis

All data are expressed as the mean ± SD (standard deviation). Statistical analyses were carried out using Prism 5.04 software (GraphPad Software). Students’ *t*-tests and one-way analysis of variance followed by Bonferroni tests were used to compare data between two groups and more than two groups, respectively. Differences were considered significant at *p* < 0.05.

## Results

### Apelin Expression Is Downregulated in AMSCs

**FIGURE 1:**
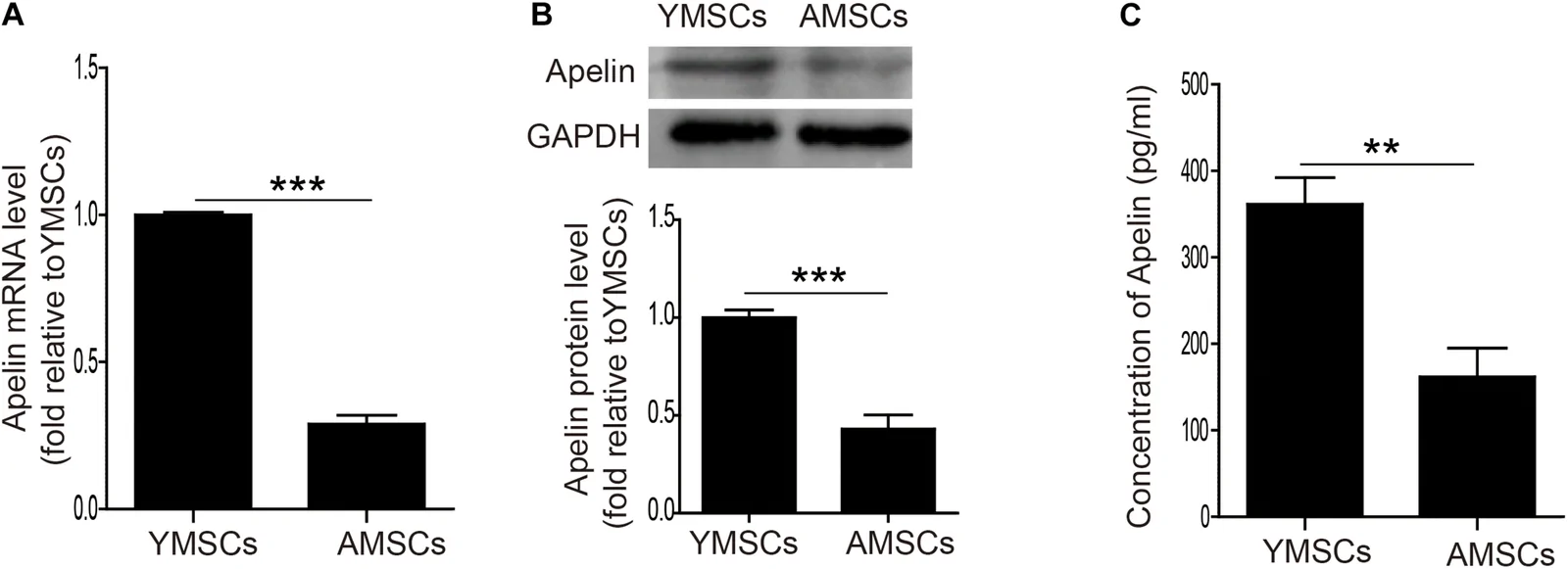
Downregulation of Apelin expression in AMSCs. **(A)** Quantitative analysis of Apelin mRNA levels in YMSCs and AMSCs. **(B)** Western blotting and quantitative analysis of Apelin protein levels in YMSCs and AMSCs. **(C)** Apelin concentrations were measured by ELISA in the CdM of YMSCs and AMSCs. Data are expressed as mean ± SD (*n* = 3). **p < 0.01, ***p < 0.001.

First, we examined Apelin expression in both YMSCs and AMSCs. Both mRNA and protein levels of Apelin were significantly downregulated in AMSCs compared with YMSCs (Figures 1A,B). Next, we measured the concentration of secreted Apelin in CdM from YMSCs and AMSCs. It was much lower in CdM from AMSCs than YMSCs (Figure 1C). These results indicate that decreased Apelin expression may affect the regulation of MSC senescence.

### Apelin Regulates MSC Senescence

**FIGURE 2:**
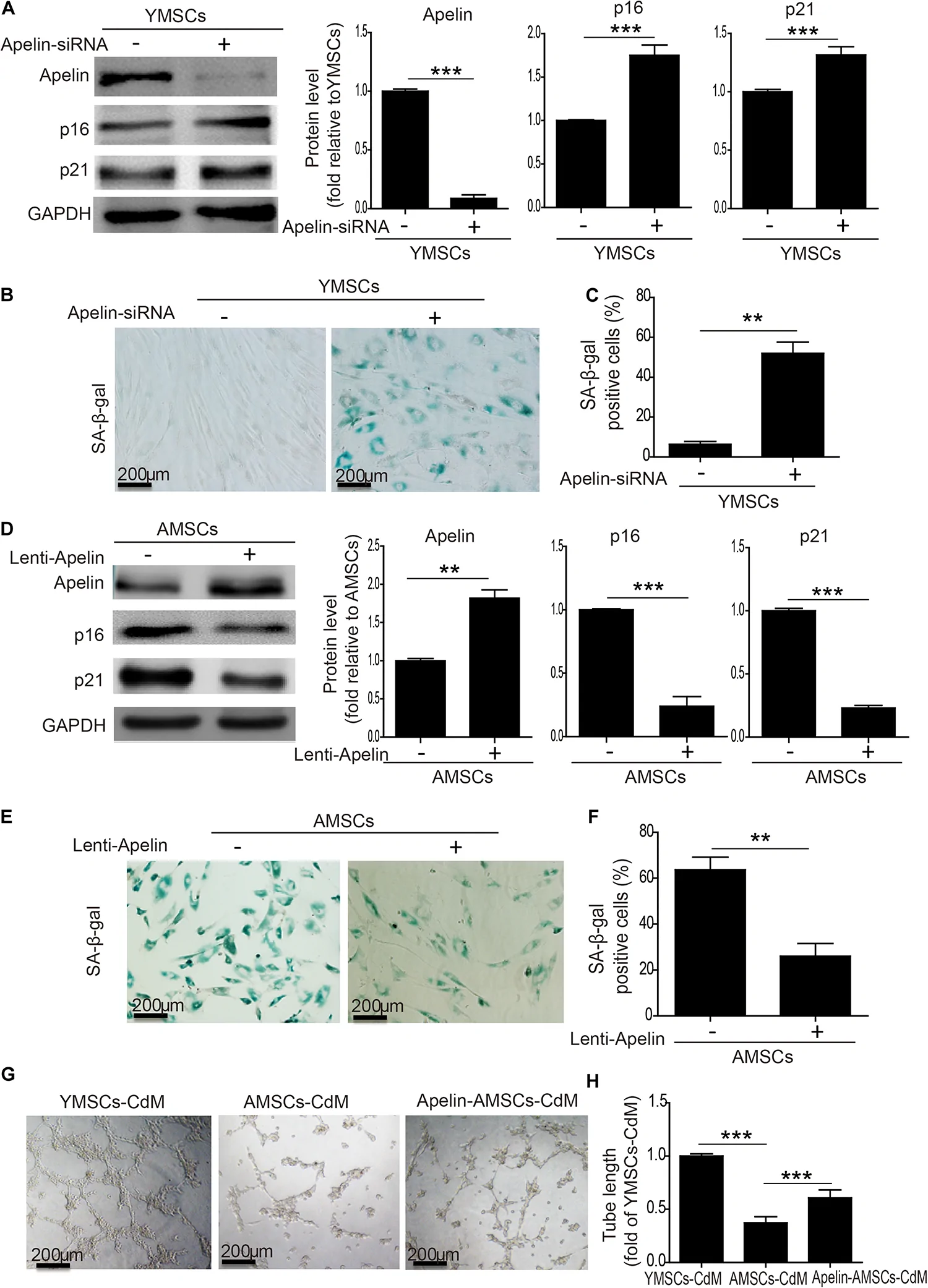
Apelin regulates MSC senescence. **(A)** Western blotting and quantitative analysis of Apelin, p16, and p12 expression in YMSCs transfected with control siRNA or Apelin-siRNA. **(B)** Representative images of SA-β-gal staining in control-siRNA and Apelin-siRNA-treated YMSCs. **(C)** Quantitative analysis of SA-β-gal-positive cells in control siRNA- or Apelin-siRNA-treated YMSCs. **(D)** Western blotting and quantitative analysis of Apelin, p16, and p12 expression in AMSCs transfected with control-lentivirus or Apelin-lentivirus. **(E)** Representative images of SA-β-gal staining in control-lentivirus- or Apelin-lentivirus-treated AMSCs. **(F)** Quantitative analysis of SA-β-gal-positive cells in control-lentivirus or Apelin-lentivirus-treated YMSCs. **(G)** Representative images of tube formation in HUVECs treated with YMSC-CdM, AMSC-CdM, or Apelin-AMSC-CdM. **(H)** Tube length analysis in HUVECs treated with YMSC-CdM, AMSC-CdM, or Apelin-AMSC-CdM. Data are expressed as mean ± SD (*n* = 3). **p < 0.01, ***p < 0.001.

To verify the role of Apelin in MSC senescence regulation, we used siRNA to knock down Apelin in YMSCs. As shown in Figure 2A, Apelin-siRNA treatment greatly decreased Apelin protein expression but increased p16 and p21 levels in YMSCs (Figure 2A). Furthermore, Apelin-siRNA treatment greatly enhanced SA-β-gal activity in YMSCs (Figures 2B,C). Next, we treated AMSCs with Apelin-lentivirus to overexpress Apelin. Apelin-lentivirus treatment increased Apelin expression and decreased p16 and p21 expression in AMSCs (Figure 2D). In addition, the number of SA-β-gal-positive cells was significantly reduced in Apelin-AMSCs compared with AMSCs (Figures 2E,F). We determined the SASP including *MMP3, IL-1*β, *RANTES* and *TNF-*α expression in YMSC, AMSC, and Apelin-AMSC by qRT-PCR (Supplementary Figure 2). The expressions of *MMP3, IL-1*β, *RANTES, TNF-*α were significantly upregulated in AMSC compared with YMSC, and Apelin overexpression in AMSC partially downregulated the expression of the above mentioned cytokines in AMSCs (Supplementary Figure 2). We next examined whether Apelin overexpression improved the paracrine effects of AMSCs. We collected CdM from YMSCs, AMSCs, and Apelin-AMSCs and evaluated their angiogenic capacities. Compared with YMSC-CdM, HUVEC tube length was significantly reduced following treatment with CdM from AMSCs. However, tube length was markedly increased in Apelin-AMSC-CdM compared with AMSC-CdM (Figures 2G,H). In accordance with tube formation, the expression levels of *FGF2, HBEGF, HGF*, and *IGF* were markedly downregulate in AMSC compared to YMSC, and Apelin overexpression partially restored the expression of the above mentioned cytokines in AMSCs (Supplementary Figure 3). Collectively, these results demonstrate that Apelin regulates MSC senescence and that its overexpression could rejuvenate AMSCs.

### Apelin Overexpression Rejuvenates AMSCs by Activating Autophagy

**FIGURE 3:**
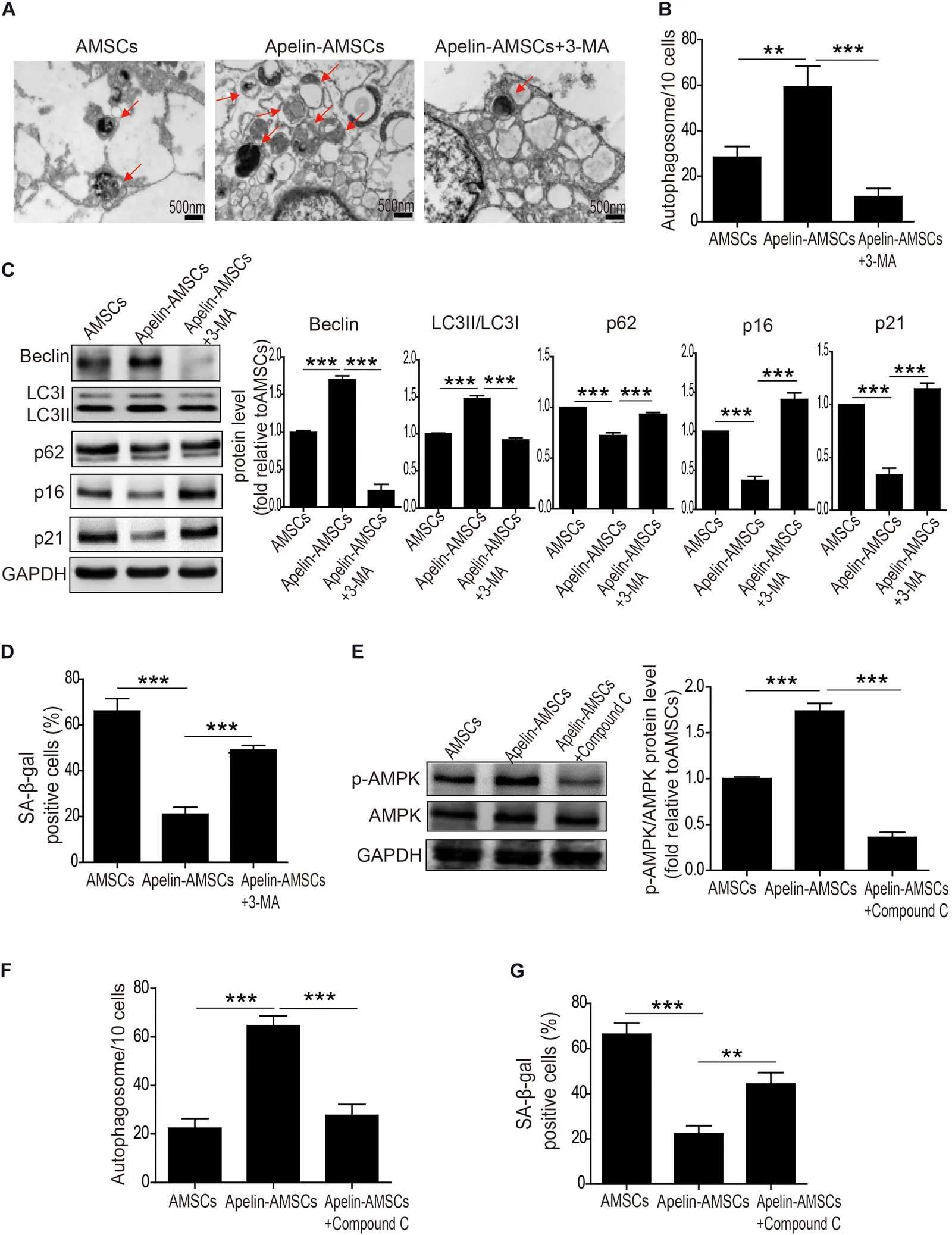
Apelin overexpression rejuvenates AMSCs by activating autophagy. **(A)** Representative TEM images of autophagosomes in YMSCs and AMSCs with or without 3-MA treatment. **(B)** Quantitative analysis of autophagosomes in AMSCs and Apelin-AMSCs with or without 3-MA treatment. **(C)** Western blotting and quantitative analysis of Beclin, LC3II/I, p62, p16, and p21 expression in AMSCs and Apelin-AMSCs with or without 3-MA treatment. **(D)** Quantitative analysis of SA-β-gal-positive cells in AMSCs and Apelin-AMSCs with or without 3-MA treatment. **(E)** Western blotting and quantitative analysis of p-AMPK and AMPK levels in AMSCs and Apelin-AMSCs with or without Compound C treatment. **(F)** Quantitative analysis of autophagosomes in AMSCs and Apelin-AMSCs with or without Compound C treatment. **(G)** Quantitative analysis of SA-β-gal-positive cells in AMSCs and Apelin-AMSCs with or without Compound C treatment. Data are expressed as mean ± SD (*n* = 3). **p < 0.01, ***p < 0.001.

We and others have shown that autophagy levels mediate MSC senescence (Ma et al., 2018; Kim et al., 2019; Zhang et al., 2019). Therefore, we examined whether Apelin overexpression rejuvenates AMSCs by regulating autophagy. It significantly increased the number of autophagosomes in AMSCs (Figures 3A,B). Apelin overexpression also upregulated protein levels of Beclin1 and LC3II/LC3I but downregulated that of p62 (Figure 3C). Combined with the decreased p16 and p21 levels and the number of SA-β-gal-positive cells in Apelin-AMSCs compared with AMSCs, we concluded that Apelin-mediated rejuvenation of aged-MSCs may be due to activation of autophagy (Figures 3C,D). However, treating Apelin-AMSCs with the autophagy inhibitor 3-methyladenine (3-MA) reduced autophagy and increased p16 and p21 levels in Apelin-AMSCs (Figures 3A–C). The number of SA-β-gal-positive cells was also greatly increased in 3-MA-treated Apelin-AMSCs compared with Apelin-AMSCs (Figure 3D). These results further confirmed that Apelin overexpression rejuvenates AMSCs by activating autophagy. Accumulating evidence indicates that AMPK signaling plays a critical role in regulating autophagy (Zhao et al., 2019; Khorraminejad-Shirazi et al., 2020). Next, we investigated whether Apelin mediates autophagy levels in AMSCs *via* this pathway. Western blotting showed that p-AMPK levels were significantly increased in Apelin-AMSCs compared with AMSCs (Figure 3E). Combined with the increased autophagy level and decreased senescence in Apelin-AMSCs (Figures 3F,G), it suggests that Apelin overexpression may activate autophagy to rejuvenate AMSCs by regulating AMPK signaling. However, treating Apelin-AMSCs with compound C (an AMPK inhibitor) robustly downregulated p-AMPK and autophagy levels and partially abrogated the reduced senescence in Apelin-AMSCs (Figures 3E–G). Collectively, these data show that Apelin overexpression activates autophagy to rejuvenate AMSCs by regulating the AMPK pathway.

### Apelin-AMSC Transplantation Improves Cardiac Function in Mice With MI

**FIGURE 4:**
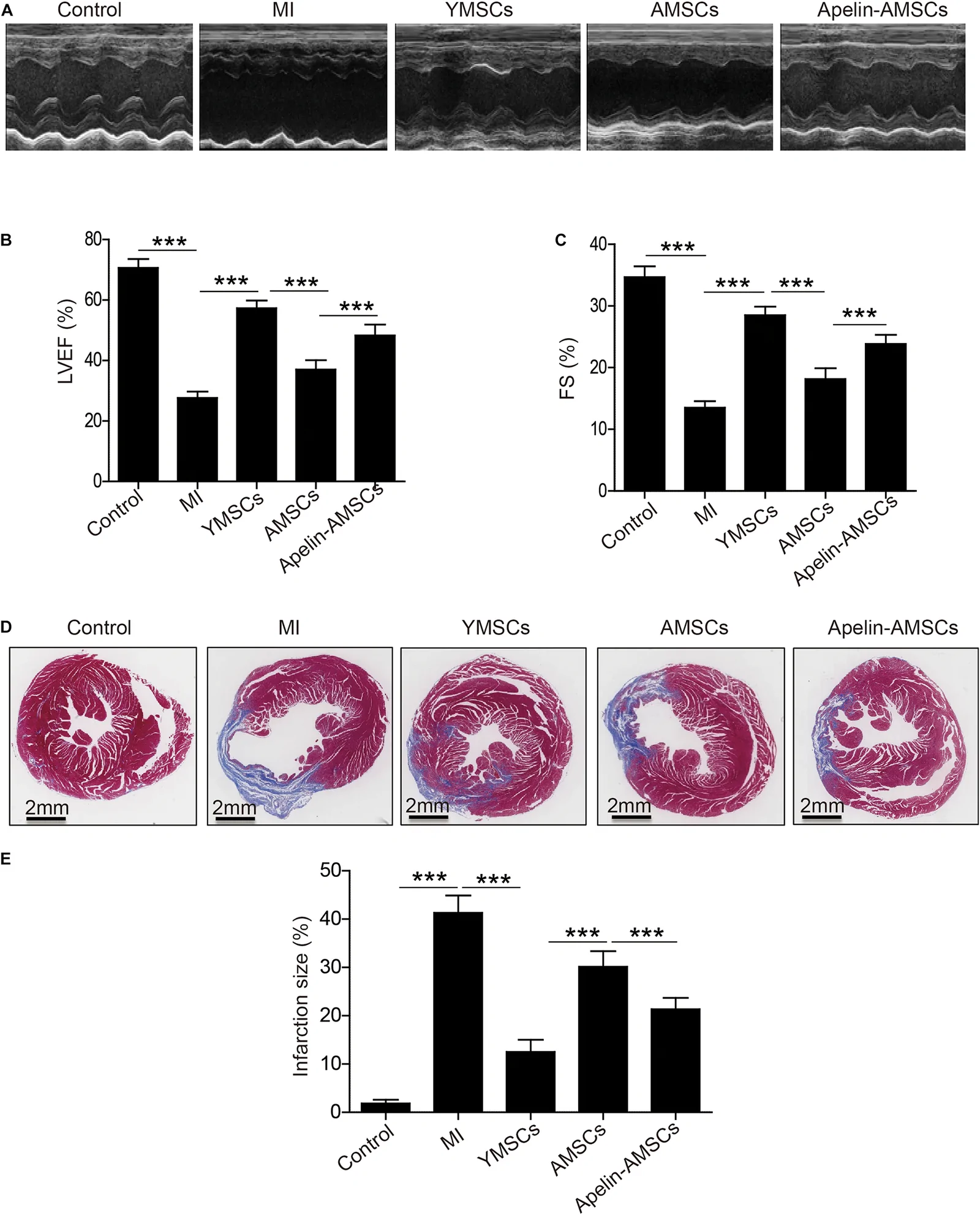
Transplantation of Apelin-aged-MSCs improves cardioprotection following infarction in mice. **(A)** Representative images of M-mode echocardiographic images captured 4 weeks following MI in mice. **(B)** Quantitative analysis of LVEF 4 weeks following MI. **(C)** Quantitative analysis of LVFS at 4 weeks following MI. **(D)** Representative images of Masson’s Trichrome staining at 4 weeks following MI. **(E)** Quantitative analysis of heart fibrosis. Data are expressed as mean ± SD (*n* = 6–7). **p < 0.01, ***p < 0.001.

To assess the therapeutic effects of Apelin-AMSCs, they were transplanted into mice following MI. Echocardiography revealed that compared with control group, left ventricle ejection fraction (LVEF) and fraction shorting (LVFS) were robustly reduced on 1 day post-Ml in the Ml group, YMSC group, AMSC group, and Apelin-AMSC group, indicating that the MI mouse model was successfully established (Supplementary Figure 4). Notably, no difference in LVEF and LVFS was observed among the MI group, YMSCs group, AMSCs group, and Apelin-AMSCs group, suggesting that a similar degree of infarction in these groups (Supplementary Figure 4). The heart function of different groups was evaluated by echocardiography 28 days after MI. Representative echocardiography images from the different groups are shown in Figure 4A. The results demonstrated that compared with the MI group, LVEF and LVFS were significantly enhanced in all MSC transplantation groups (Figures 4B,C). Although LVEF and LVFS were the highest in the YMSC group 28 days after cell transplantation, both values were much higher in the Apelin-AMSC group compared with the AMSC group (Figures 4B,C). Next, we examined infarction size using Masson’s trichrome staining. Compared with the MI group, infarction size was significantly decreased in all MSC transplantation groups (Figures 4D,E). Notably, the YMSC group had smaller infarcts than the Apelin-AMSC and AMSC groups (Figures 4D,E). Furthermore, infarct size in the Apelin-AMSC group was greatly reduced compared to the AMSC group (Figures 4D,E). Taken together, these data indicate that Apelin-AMSCs have greater therapeutic efficacy for MI.

### Apelin Overexpression Improves AMSC Survival in the Ischemic Mouse Heart Following MI

**FIGURE 5:**
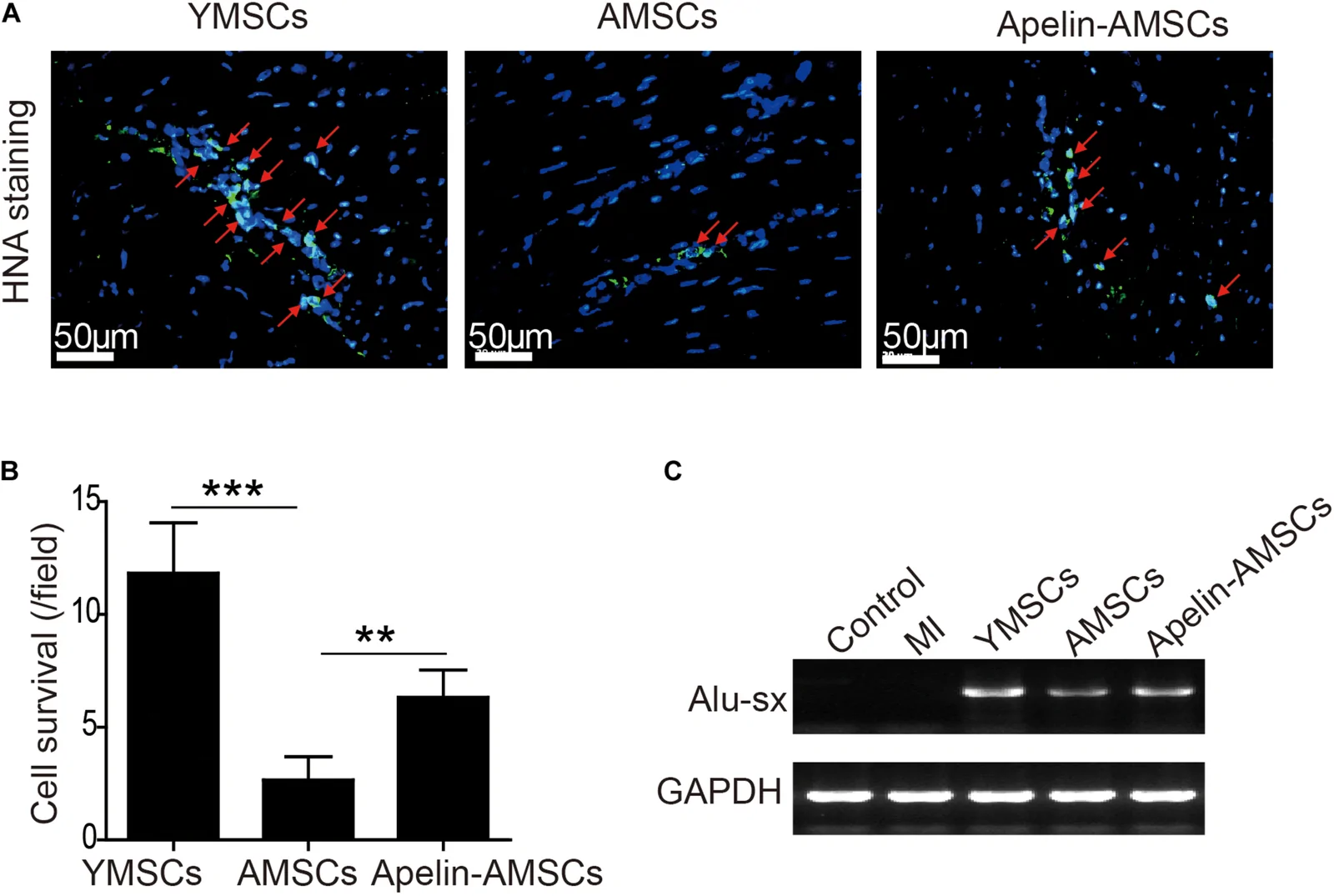
Apelin overexpression improves AMSC survival in the ischemic mouse heart after MI. **(A)** Representative images of anti-HNA-positive cells in ischemic hearts 4 weeks after MSC transplantation. **(B)** Quantitative analysis of MSC survival in ischemic mouse hearts 4 weeks after MI. **(C)** Alu-sx expression was examined by PCR in the YMSC, AMSC, and Apelin-AMSC transplantation groups. Data are expressed as mean ± SD (*n* = 6). **p < 0.01, ***p < 0.001.

Since the transplanted MSCs were isolated from human, we first performed anti-HNA staining to detect MSC survival 28 days after transplantation. MSCs were detected in all three MSC-transplanted groups (Figure 5A). The number of surviving MSCs in ischemic heart tissue was significantly higher in the YMSC group than in the AMSC and Apelin-AMSC groups. Notably, the number of surviving MSCs in the Apelin-AMSC group was much higher than in the AMSC group (Figure 5B). Next, we performed PCR for the human repeat sequences Alu-sx to further evaluate MSC survival in heart tissue. As Alu-sx was detected in all MSC-transplanted groups, but not in the sham or MI group (Figure 5C). Although Alu-sx expression was highest in the YMSC group, it was much higher in the Apelin-AMSC group compared to the AMSC group (Figure 5C). These results indicate that Apelin overexpression could enhance AMSC tolerance to ischemic challenges.

### Apelin-AMSC Transplantation Enhances Angiogenesis in the Ischemic Mouse Heart

**FIGURE 6:**
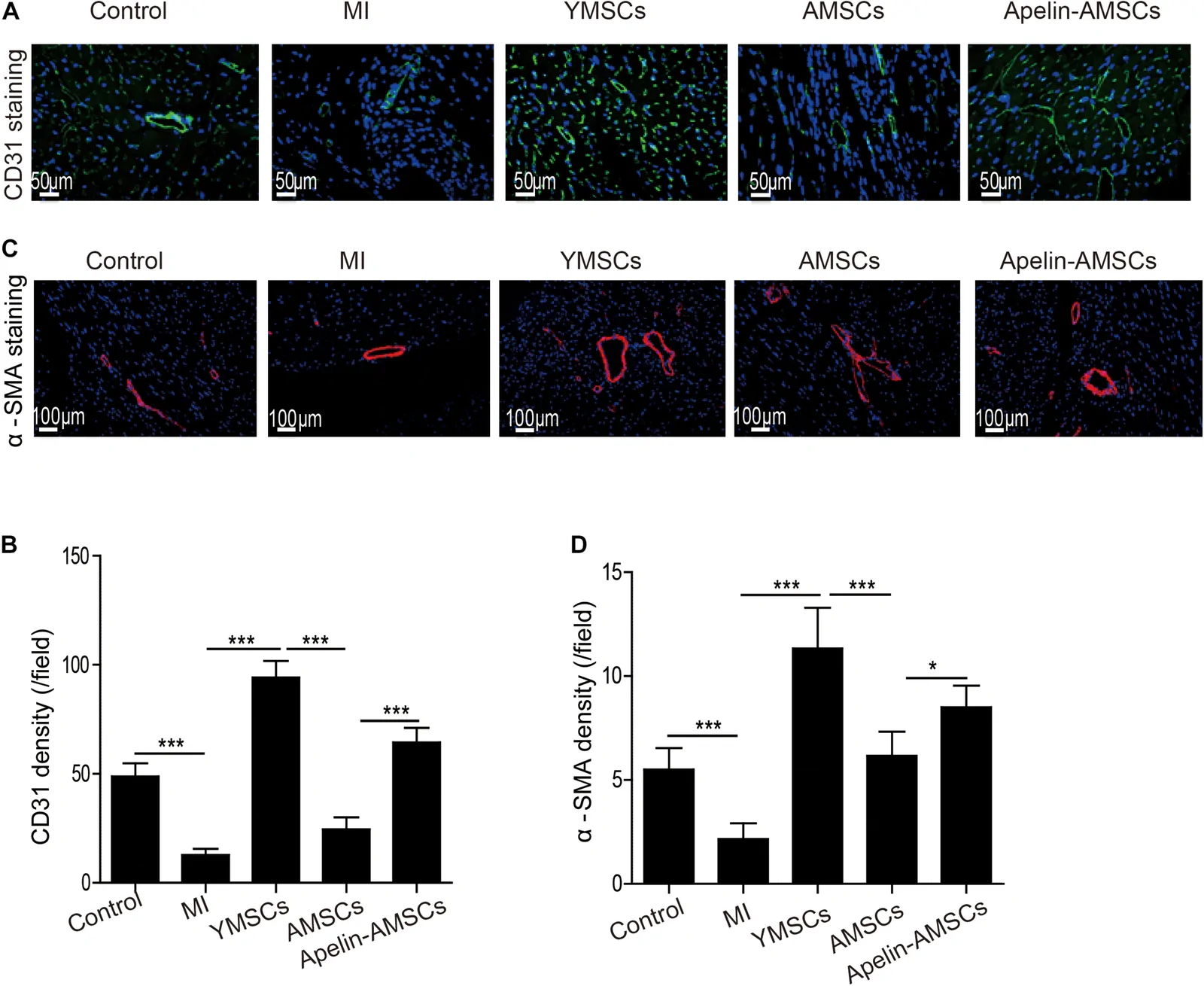
Apelin-AMSC transplantation enhances angiogenesis in the ischemic mouse heart after MI. **(A)** Representative images of CD31 staining in ischemic mouse hearts 28 days following MI. **(B)** Quantitative analysis of CD31 density in ischemic hearts 28 days following MI. **(C)** Representative images of α-SMA staining in ischemic hearts 28 days following MI. **(D)** Quantitative analysis of α-SMA density in ischemic hearts 28 days following MI. Data are expressed as mean ± SD (*n* = 6). **p < 0.01, ***p < 0.001.

Next, we examined that whether Apelin-AMSC transplantation could enhance angiogenesis in the ischemic mouse heart. The capillary densities in ischemic heart tissue from different groups was detected by CD31 staining. Compared with the control group, CD31-positive capillary density was significantly reduced in the MI group but increased in the MSC-transplanted groups (Figures 6A,B). Notably, the YMSC group had higher capillary density than the Apelin-AMSC and AMSC groups (Figures 6A,B). Furthermore, the capillary density in the Apelin-AMSC group was much higher than in the AMSC group (Figures 6A,B). A similar result was observed when we examined arteriole densities in the different MSC-transplanted groups. Arteriole density was dramatically enhanced in all MSC-transplanted groups compared with the MI group, and it was highest in the YMSC group (Figures 6C,D). Notably, the Apelin-AMSC group had a much higher arteriole density than the AMSC group (Figures 6C,D). Collectively, these results demonstrate that Apelin-AMSC transplantation enhances angiogenesis in the infarcted mouse heart.

## Discussion

There were several major findings in this study. First, Apelin expression was remarkably reduced in AMSCs. Second, downregulation of Apelin inhibited AMPK signaling and induced MSC senescence by regulating autophagy. Third, Apelin overexpression rejuvenated AMSCs and enhanced their paracrine effects. Finally, compared with AMSCs, transplantation of Apelin-AMSCs had greater beneficial effects in mice after MI because they improved cell survival and angiogenesis.

MSC-based therapy has been intensively investigated as a potential treatment for MI for many years (Zhang et al., 2015; Faiella and Atoui, 2016; Yan et al., 2020). Despite promising results of MSC-based therapy for MI in animal studies and clinical trials, MSC senescence is a major contributor that diminishes the regenerative potential. The number and function of MSCs dramatically decline in physiologically aged individuals and aging-associated patients (Hu et al., 2020). Although autologous MSC transplantation avoids immunorejection issues, most MI patients are elderly, and transplantation of old autologous MSCs fails to achieve satisfactory results (Trounson and McDonald, 2015; Zhang D. Y. et al., 2020). Compared with YMSCs, AMSCs are more sensitive to oxidative stress, so they have decreased survival capacity in the ischemic heart tissue (Zhang D. Y. et al., 2020). AMSCs also have decreased paracrine effects, especially with regard to angiogenic capacity. Therefore, it is of great value to identify the key molecules that govern MSC senescence. Apelin is an adipokine that is widely expressed in mammalian cells including MSCs (Zeng et al., 2012) and vascular smooth muscle cells (Wang et al., 2019). Apelin is reportedly involved in regulating various biological effects including immunological integration, cell homeostasis, and cardiovascular activities (Foroughi et al., 2019). It can also effectively increase MSC survival under hypoxic-ischemic challenge *in vitro*, indicating that Apelin plays a pivotal role in regulating MSC biology (Hou et al., 2017). In the current study, we found that Apelin expression was remarkably reduced in AMSCs compared with YMSCs, suggesting that Apelin level may be associated with MSC senescence. Subsequently, we revealed the essential role of Apelin in MSC senescence regulation using loss-gain-of-function approaches. Apelin overexpression in AMSCs ameliorated MSC senescence and enhanced cell survival after transplantation in a mouse MI model. Consistent with previous studies reporting that Apelin mediates cellular angiogenesis, overexpression of Apelin enhanced AMSC angiogenic capacity as evidenced by the longer tube length CdM-stimulated HUVECs. Notably, transplantation of Apelin-AMSCs improved the capillary density of the ischemic areas compared with AMSCs. Collectively, our results indicate that enhancing Apelin levels rejuvenates AMSCs and have promising therapeutic effects following MI.

Autophagy is a process that sequesters injured cytoplasmic organelles and impaired macromolecules into autophagosomes that are transferred into lysosomes for degradation. Although the underlying mechanisms remain unclear, increasing evidence suggests that autophagy deficiency contributes to MSC senescence (Infante et al., 2014; Zhang D. et al., 2020). Ma et al. (2018) found that autophagy is significantly reduced in AMSCs compared with YMSCs, and 3-MA treatment induces YMSCs into a relatively aged state as manifested by deceased proliferation capacity and osteogenic differentiation. Previous studies have documented that Apelin regulates autophagy (Xie et al., 2015; Chen et al., 2020), thus it is important to clarify whether Apelin is a key molecule that regulates MSC senescence by mediating autophagic activity. Here we found that Apelin overexpression increased autophagic activity, as evidenced by enhanced expression of Beclin and LC3II/I and decreased expression of p62. This was due to inhibition of AMPK signaling pathway and rejuvenated cellular senescence in AMSCs, and these effects were partially reversed by 3-MA or compound C treatment. Given that autophagy mediates MSC senescence, activation of autophagy may be a novel strategy to rejuvenate senescent MSCs. A combination of AICAR (an AMPK stimulator) and nicotinamide (a sirtuin1 activator) was shown to synergistically improve the proliferative capacity of MSCs and ameliorate the aged phenotype by promoting autophagic activity (Khorraminejad-Shirazi et al., 2020). Similarly, Apelin overexpression attenuated senescence-associated changes in AMSCs by activating AMPK signaling to enhance autophagy.

Our study also has some limitations that must be acknowledged. First, although we found that Apelin regulates physiological senescence in AMSCs, whether it mediates MSC replicative senescence during long-term *in vitro* culture has not been determined. Second, since Apelin is involved in regulating multiple pathways in addition to autophagy, whether Apelin mediates MSC senescence *via* other signaling mechanisms requires further investigation. Third, exosomes serve as paracrine mediators and play a critical role in MSC-mediated cardioprotection, whether Apelin could enhance the beneficial effects of exosomes for MI is unknown. Forth, the transplanted MSCs are still detectable at day 28. A longer observation period might provide more information of Apelin-MSC-mediated protective effects. Finally, although no side effects were observed in MI model mice that underwent Apelin-AMSC transplantation, the genomic stability of Apelin-AMSCs must be carefully evaluated before their clinical application.

## Conclusion

In summary, our study revealed that Apelin plays an essential role in regulating MSC senescence by mediating autophagy. Apelin overexpression rejuvenated AMSCs and enhanced cardiac protection following infarction by improving cell survival and activating angiogenesis. We identified Apelin as a novel regulator for rejuvenating AMSCs that could enhance the therapeutic efficacy of autologous AMSC transplantation for MI.

## Data Availability Statement

The raw data supporting the conclusions of this article will be made available by the authors, without undue reservation.

## Ethics Statement

The animal study was reviewed and approved by the Committee on the Use of Live Animals in Teaching and Research of the Tongji University for Laboratory Animal Medicine.

## Author Contributions

YZ and XLia designed the research, analyzed the data, and wrote the manuscript. HaZ and CZ performed the research, analyzed the data, and wrote the manuscript. GJ, BH, HuZ, YH, ZC, LS, FL, and LW contributed to performing the research and preparing experimental reagents. XLi, YD, and LW helped analyze the data and provided the materials. All authors contributed to the article and approved the submitted version.

## Conflict of Interest

The authors declare that the research was conducted in the absence of any commercial or financial relationships that could be construed as a potential conflict of interest.
