# Supplementary material for: Apelin Rejuvenates Aged Human Mesenchymal Stem Cells by Regulating Autophagy and Improves Cardiac Protection After Infarction
Source: Front Cell Dev Biol. 2021 Mar 2;9:628463. doi: 10.3389/fcell.2021.628463 (PMC7960672; doi:10.3389/fcell.2021.628463)
Supplement: Supplementary file 1 [file Data_Sheet_1.PDF]

Supplementary Information

Apelin Rejuvenates Aged Human Mesenchymal Stem Cells by Regulating Autophagy and Improves Cardiac Protection After Infarction

Hao Zhang[1,2#], Chenling Zhao[3#], Guojun Jiang[1], Bei Hu[2], Huifeng Zheng[2], Yimei Hong[2], Zhen Cui[4], Linli Shi[2], Xin Li[2], Fang Lin[5,6], Yue Ding[7], Lu Wei[6], Mimi Li[6], Xiaoting Liang[5,6*], Yuelin Zhang[1,2*]

[1]Faculty of Pharmacy, Bengbu Medical College, Bengbu, Anhui, P.R. China;

[2]Department of Emergency Medicine, Department of Emergency and Critical Care Medicine, Guangdong Provincial People's Hospital, Guangdong Academy of Medical Sciences, Guangzhou, Guangdong, P.R. China;

[3]Department of Respiratory Medicine, the First Affiliated Hospital of Bengbu Medical College, Bengbu, Anhui, P.R China;

[4]Department of Radiation Oncology, the First Affiliated Hospital of Bengbu Medical College, Bengbu, Anhui, P.R. China;

[5]Institute of Regenerative Medicine, Shanghai East Hospital, Tongji University School of Medicine, Shanghai, P.R. China;

[6]Clinical Translational Medical Research Center, Shanghai East Hospital, Tongji University School of Medicine, Shanghai, P.R. China;

[7]Department of Organ Transplantation, Changzheng Hospital, Second Military Medical University, Shanghai, P.R. China

*Address correspondence to:

Dr. Xiaoting Liang, MD, PhD; Institute of Regenerative Medicine, Shanghai East Hospital, Tongji University School of Medicine, Shanghai, China

Email: liangxt@tongji.edu.cn

Dr. Yuelin Zhang, MD, PhD;

Faculty of Pharmacy, Bengbu Medical College, Bengbu, Anhui, China

E-mail: zhangyuelin1999@163.com

Figure legend

Supplementary Figure 1. Structures of lentiviral plasmid using for Apelin overexpression.

Supplementary Figure 2. Expression of SASP genes including *MMP3, IL-1β, RANTES* and *TNF-α* determined by qRT-PCR. Data are expressed as mean±SD (n=3~4). *$p<0.05$, **$p<0.01$, ***$p<0.001$.

Supplementary Figure 3. Expression of pro-angiogenesis genes including *FGF2, HBEGF, HGF* and *IGF* determined by qRT-PCR. Data are expressed as mean±SD (n=3~4). *$p<0.05$, ***$p<0.001$.

Supplementary Figure 4. Quantitative analysis of LVEF and LVFS at 1 day following MI. Data are expressed as mean±SD (n=6~7).
