# Supplementary figures and images for: Apelin Rejuvenates Aged Human Mesenchymal Stem Cells by Regulating Autophagy and Improves Cardiac Protection After Infarction
Source: Front Cell Dev Biol. 2021 Mar 2;9:628463. doi: 10.3389/fcell.2021.628463 (PMC7960672; doi:10.3389/fcell.2021.628463)

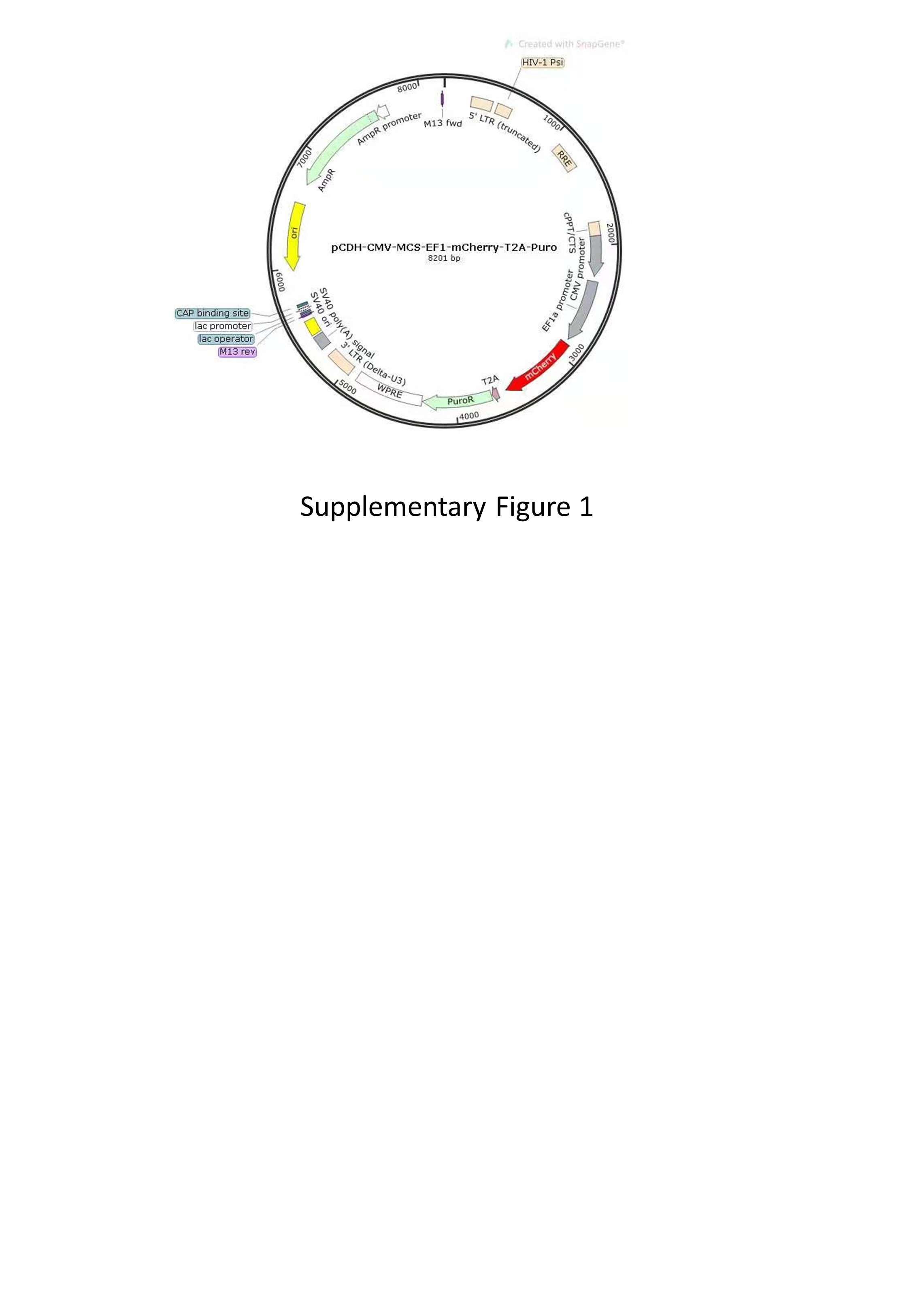

Supplement: Supplementary file 2 [file Image_1.JPEG]

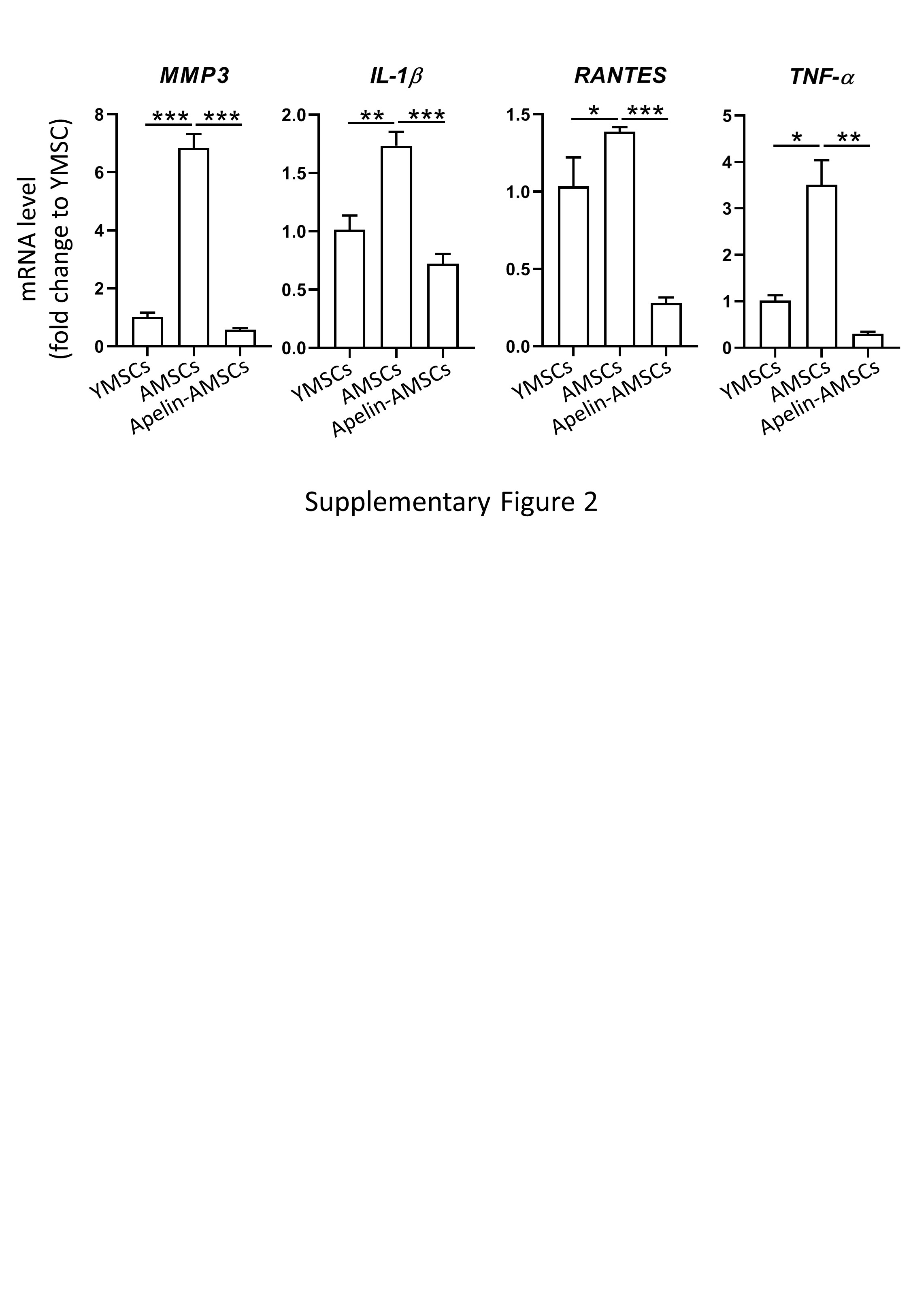

Supplement: Supplementary file 3 [file Image_2.JPEG]

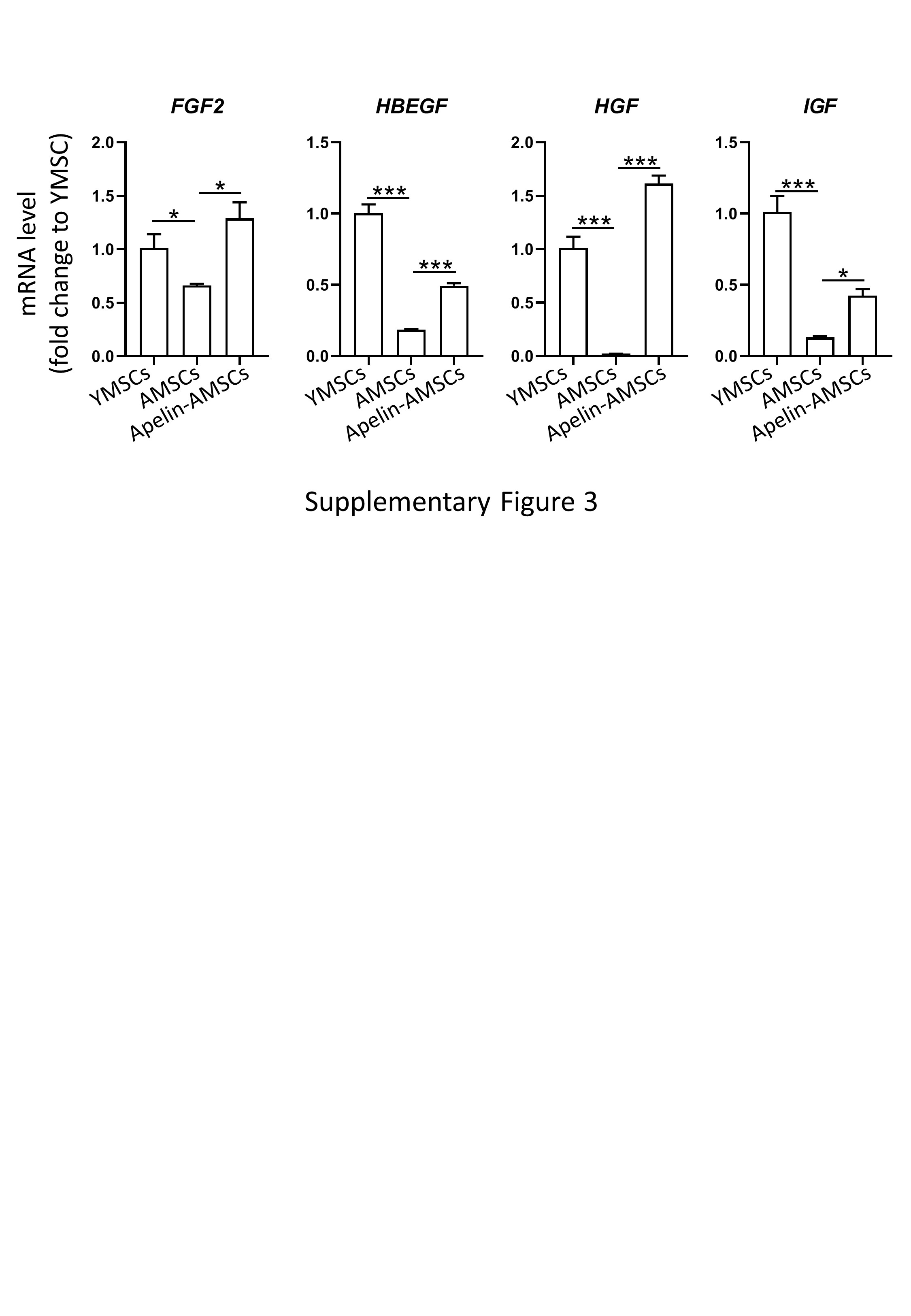

Supplement: Supplementary file 4 [file Image_3.JPEG]

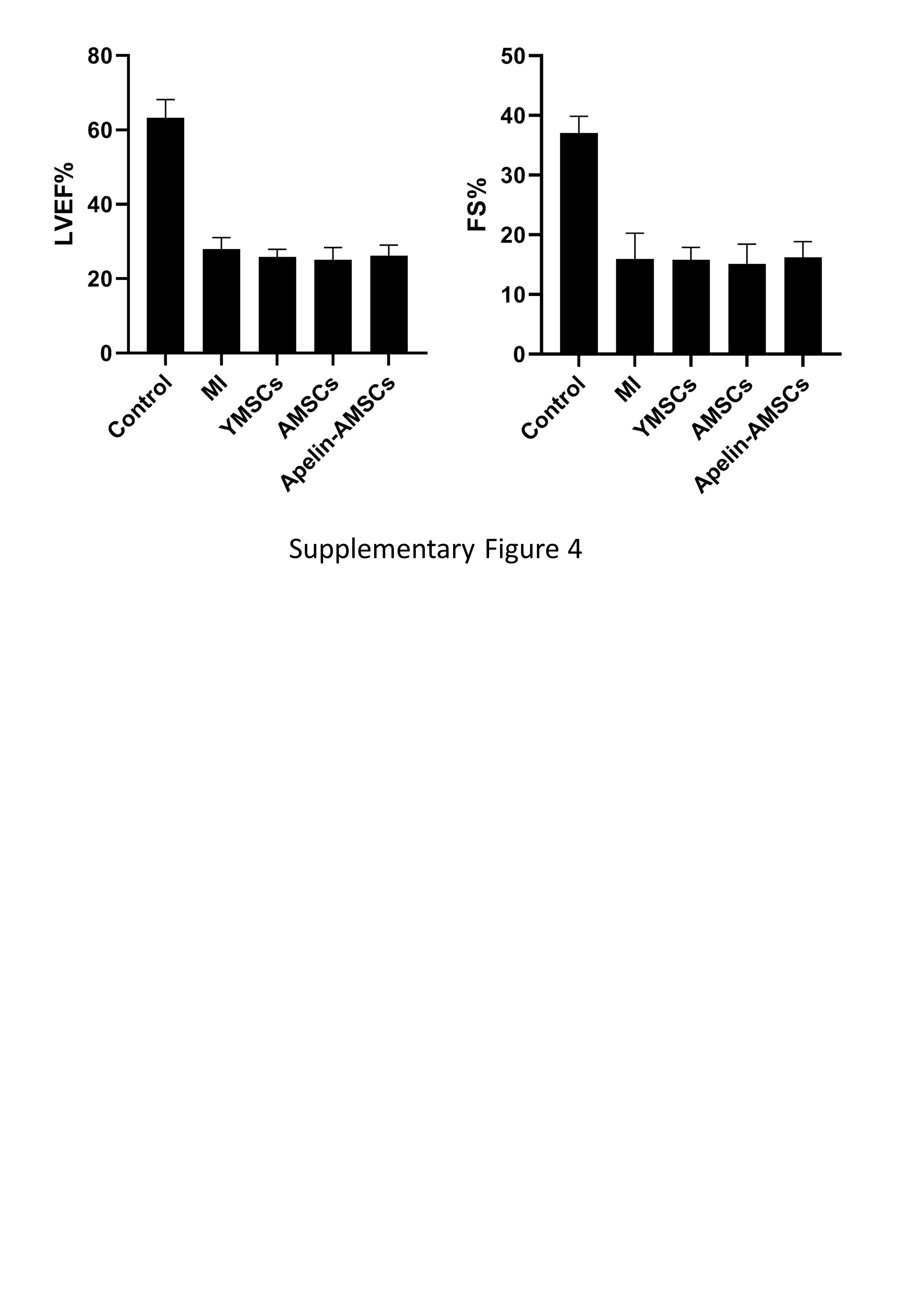

Supplement: Supplementary file 5 [file Image_4.JPEG]
